# Determinants of hypothermia on neonates admitted to the intensive care unit of public hospitals of Central Zone, Tigray, Ethiopia 2017: unmatched case–control study

**DOI:** 10.1186/s13104-018-3691-0

**Published:** 2018-08-13

**Authors:** Hagos Tasew, Kahsu Gebrekristos, Kalayu Kidanu, Teklewoini Mariye, Girmay Teklay

**Affiliations:** 1 Department of Nursing, College of Health Science, Aksum University, Aksum, Ethiopia; 2 Department of Nursing, College of Health Science, Mekelle University, Mekelle, Ethiopia

**Keywords:** Hypothermia, Determinants, Neonates, Central Zone, Tigray, Ethiopia

## Abstract

**Objective:**

This study aims to identify determinants of hypothermia in neonates in neonatal intensive care unit of public hospitals of Central Zone Tigray, Ethiopia in 2017.

**Results:**

A total of 88 cases and 176 controls were included in this study. Ninety-one percent cases and 86.4% controls were in the 1st week of neonate age. Multivariable logistic regression analysis showed that delayed initiation of breastfeeding [AOR = 7.23; 95% CI (2.75, 18.99)], low birth weight [AOR = 8.51; 95% CI (2.71, 26.73)], preterm [AOR = 3.689; 95% CI (1.359, 10.012)], low APGAR score at 5th min [AOR = 3.71; 95% CI (1.57, 8.79)], skin to skin contact [AOR = 6.23; 95% CI (2.523, 15.358)], night time delivery [AOR = 6.25; 95% CI (2.58, 15.12)] and bathed within 24 h [AOR = 10.06; 95% CI (3.86, 26.22)] were independent risk factors of neonatal hypothermia.

## Introduction

Neonatal hypothermia is subnormal of a newborn’s body temperature drops below 36.5 °C [1]. It leads to diverse health consequences with a prevalence in hospitals ranges from 32 to 85% and homes ranges from 11 to 92%, including tropical environments [2]. Immediately after delivery if no action is taken, the core and skin temperatures of term neonates can decrease at a rate of approximately 0.1 °C and 0.3 °C per minute respectively. The mechanisms of heat loss in neonates could be through conduction, convection, evaporation, and radiation immediately following after birth [3].

Even though there are guidelines in place for managing hypothermia, thermal care and has included thermal care as one of the elements of essential newborn care that should be provided to all newborns regardless of setting [1, 4], hypothermia is one of the major contributor to neonatal morbidity and mortality in low and middle income countries. In addition, in poor settings, only limited progress has been made towards understanding the risk of mortality by hypothermia [5]. In several resource-limited countries, some progress has been attained in subsiding mortality of children under 5 years of age, but less progress has been made to increase subsistence of neonates or infants under the age of 28 days [6]. Consequently, lack of thermal protection is still an underappreciated major challenge for newborn survival in developing countries [2].

To solve the major challenge of morbidity and mortality of neonatal hypothermia needs to identify its determinants, which have greater input to program managers and policy makers for designing, proper implementation and evaluation of programs on reduction of neonatal mortality and improvement of newborn care to achieve sustainable development goal (SDG) 3 of ensuring healthy lives and promote well- being for all at all age. In this article, we have quantified associations between neonatal hypothermia and determinants.

## Main text

### Study design

A hospital-based unmatched retrospective case–control study design was employed on neonates in the neonatal intensive care unit of the Central Zone, Tigray, Ethiopia 2017.

### Source of population

The source population was all neonates paired mothers admitted to the neonatal intensive care unit of public hospitals in the study area during the study period. Cases were neonates with hypothermia (< 36.5 °C) [1] admitted to the neonatal intensive care unit of four public hospitals in the Central Zone. Controls were neonates without hypothermia or ≥ 36.5 °C admitted at NICU in the same health institutions of cases.

### Sample size determination

A sample size of the study was calculated using Epi-info software version 7.1.1 with the following parameters for unmatched case–control study. The parameters were significance 95%, power 80%, odds ratio 2.62, case to control ratio 1: 2, the proportion of controls with exposure 15.5% [7], and proportion of cases with exposure = 36.71% and got 80 cases and 160 controls After assuming a 10% non-response rate, the total sample size yield 88 cases and 176 controls with the overall sample size were 264.

### Sampling technique

Consecutive sampling technique was used to select the study subjects from four public hospitals (Aksum University referral hospital, St. Marry hospital, Adwa hospital and Abyiadi hospital).

### Data collection tool and procedure

The questionnaire was initially prepared in English and then translated into local language Tigrigna. Data were collected from the mothers, neonates, and chart of the neonates using interviewer-administered structured questionnaire adopted and modified from different kinds of literature and observational. Four BSc nurses’ data collectors with previous experience of data collection were recruited to run the data collection procedure. Continuous follow-up and supervision were made by supervisors and principal investigators throughout the data collection period. Data collection was accomplished within 6 weeks’ duration, starting from February to March 2017. A digital thermometer was used to identify cases and controls, which measures the surface temperature at the site of axilla according to WHO recommendation [1]. Ten percent pretest was done at Suhual hospital North West Tigray to check the consistency of the questioner.

### Data analysis and process

Data were cleaned and entered using Epi info version 7.1.1 and analyzed using SPSS version 22.0 statistical software. Multivariable logistic regression was done to determine determinants of hypothermia. The goodness of fit of the model was checked using Hosmer–Lemen show test. All assumptions of logistic regression were checked. Collinearity of the variables was checked variance inflation factors (VIF) and tolerance. If VIF < 10 and tolerance > 0.1 indicating that no multicollinearity among variables. Data was finally presented and interpreted at P-value < 0.05 were considered as statistically significant in multivariable logistic regression.

### Results

#### Socio-demographic characteristics of mothers

In this study, a total of 88 neonates who had hypothermia (cases) with their index mothers and 176 neonates who had no hypothermia (controls) with their index mothers were included, making a response rate of 100%. The median (± inter quartile range) age of mothers was 27.5 ± 8 years’ ranges from 18 to 44 years. Forty-six (53.3%) of cases of mothers and 71 (40.3%) controls of mothers were living in rural areas. Most mothers 69 (78.4%) cases and 141 (80.1%) controls were orthodox. Regarding marital status, 79 (89.8%) cases of mothers and 167 (94.9%) controls of mothers were married. Twenty-nine (32.9%) of cases of mothers and 81 (46.0%) controls of mothers were housewives by occupation and 27 (30.7%) cases of mothers and 52 (29.5%) controls of mothers were not able to read and write. Concerning neonates’ socio-demographic characteristics, 80 (90.9%) of the cases and 152 (86.4%) controls were found below the age of 7 days.

#### Obstetric history of mother’s characteristics of the study subjects

In this study, 45 (51.1%) cases of mothers and 79 (44.9%) controls of mothers were prim parous and 27 (30.7%) cases of mothers and 42 (23.9%) controls of mothers had complications during pregnancy and labor. Fourteen (15.9%) cases and 15 (8.5%) controls were delivered by Caesarian section. Twenty-one (23.9%) cases and 32 (18.2%) controls were delivered at home whereas 24 (27.3%) cases and 75 (42.6%) controls were delivered in hospital (Table 1).

**Table 1:** Obstetric history and practice of mothers in Central Zone Tigray, Ethiopia, 2017

Variables	Category	Cases	Controls	Total
		n = 88 (%)	n = 176 (%)	n = 264 (%)
Parity	Prim parous	45 (51.1)	79 (44.9)	124 (47.0)
	Multiparous	43 (48.9)	97 (55.1)	140 (53.0)
Obstetric complication	Yes	27 (30.7)	42 (23.9)	69 (26.1)
	No	61 (69.3)	134 (76.1)	195 (73.9)
Mode of delivery	Spontaneous	65 (73.9)	142 (80.7)	207 (78.4)
	Instrumental	9 (10.2)	19 (10.8)	28 (10.6)
	C/s	14 (15.9)	15 (8.5)	29 (11.0)
Place of delivery	Hospital	24 (27.3)	75 (42.6)	99 (37.5)
	Health center	42 (47.7)	69 (39.2)	111 (42.1)
	Private clinic	1 (1.1)	0 (0)	1 (0.4)
	Home	21 (23.9)	32 (18.2)	53 (20.0)
Type of pregnancy	Single	80 (90.9)	169 (96.0)	249 (94.3)
	Twin	8 (9.1)	7 (4.0)	15 (5.7)
Time of delivery	Day	27 (30.7)	101 (57.4)	128 (48.5)
	Night	61 (69.3)	75 (42.6)	136 (51.5)
Starting breastfeeding	Immediately	19 (21.6)	90 (51.1)	109 (41.3)
	Within 1 h	16 (18.2)	36 (20.5)	52 (19.7)
	After 1 h	53 (60.2)	50 (28.4)	103 (39.0)

Obstetric complication comprises any complication during pregnancy, labor and after labor

#### Neonate, environmental and practice related characteristics

The mean (± SD) room temperature of the neonatal intensive care unit was 29.01 ± 1.30 °C. Thirty (34.1%) cases and 18 (10.2%) controls had low birth weight and 61 (69.3%) cases and 156 (88.6%) controls were delivered at term gestational age. Forty-nine (55.7%) cases and 36 (20.5%) controls were bathed their child within 24 h of delivery. Forty-eight (54.5%) cases and 151 (85.8%) controls were done skin to skin contact immediately after delivery (Table 2).

**Table 2:** Neonatal, environment and practice related characteristics in Central Zone Tigray, Ethiopia, 2017

Variables	Category	Cases	Controls	Total
		n = 88 (%)	n = 176 (%)	n = 264 (%)
Weight of neonate	< 2500 g	30 (34.1)	18 (10.2)	48 (18.2)
	≥ 2500 g	58 (65.9)	158 (89.8)	216 (81.8)
Gestational age	< 37 weeks	27 (30.7)	20 (11.4)	47 (17.8)
	≥ 37 weeks	61 (69.3)	156 (88.6)	217 (82.2)
Bathing in 24 h	Yes	49 (55.7)	36 (20.5)	85 (32.2)
	No	39 (44.3)	140 (79.5)	179 (67.8)
APGAR at 5 min	< 7	48 (54.5)	43 (24.4)	91 (34.5)
	≥ 7	40 (45.5)	133 (75.6)	173 (65.5)
Skin to skin contact	Yes	48 (54.5)	151 (85.8)	199 (75.4)
	No	40 (45.5)	25 (14.2)	65 (24.6)
CPR	Yes	18 (20.5)	30 (17.0)	48 (18.2)
	No	70 (79.5)	146 (83.0)	216 (81.8)

#### Determinants of hypothermia

The majority of variables which were showed significant association with risk of neonatal hypothermia in the bivariate analysis could not persist as significant in the multivariable logistic regression analysis. In the multivariable binary logistic regression analysis, only seven variables had shown an overall significant effect on the risk of neonatal hypothermia at 5% level of significance.

Hence, mothers bathed their child within 24 h were showed significant association with neonatal hypothermia. The odds of bathed their child within 24 h were 10 times higher compared to those who have not bathed their child within 24 h [AOR = 10.06; 95% CI (3.86, 26.22)].

Lack of skin to skin contact after delivery right away was significantly associated with neonatal hypothermia. This study showed that those who were not experiencing skin to skin contact 6 times higher risk than those who had experienced skin to skin contact to neonatal hypothermia [AOR = 6.23; 95% CI (2.52, 15.36)]. Mothers started breastfeeding their child after 1 h had 7 times higher risk than those who started immediately after born to the outcome of neonatal hypothermia [AOR = 7.23; 95% CI (2.75, 18.99)].

APGAR score at five minutes had a significant association with the outcome variable of neonatal hypothermia. Those less than seven scores were 3.7 times higher risk than greater than seven scores to neonatal hypothermia [AOR = 3.71; 95% CI (1.57, 8.79)]. The odds of night delivered were 6 times higher at risk than daytime delivered for neonatal hypothermia [AOR = 6.252; 95% CI (2.58, 15.12)].

Preterm babies were 3.6 times higher risk than term developing neonatal hypothermia [AOR = 3.67; 95% CI (1.34, 10.01)]. Similarly, the weight of the neonate had also a significant association with neonatal hypothermia. Low birth weight neonates were 8.5 times higher at risk than normal weight as a determinant of neonatal hypothermia [AOR = 8.51; 95% CI (2.71, 26.73)] (Table 3).

**Table 3:** Predictors of hypothermia on neonates in hospitals of Central Zone Tigray, Ethiopia, 2017

Variables	Category	Cases	Controls	COR [95% CI]	AOR [95% CI]
		n = 88 (%)	n = 176 (%)		
Residence	Urban	42 (47.7)	105 (59.7)	1	1
	Rural	46 (53.3)	71 (40.3)	1.620 (0.967, 2.712)	1.308 (0.500, 3.425)
Bathing in 24 h	Yes	49 (55.7)	36 (20.5)	4.886 (2.797, 8.534)	10.06 (3.862, 26.219)^a^
	No	39 (44.3)	140 (79.5)	1	1
Skin to skin contact	Yes	48 (54.5)	151 (85.8)	1	1
	No	40 (45.5)	25 (14.2)	5.033 (2.774, 9.134)	6.225 (2.523, 15.358)^a^
Initiation of breastfeeding	Immediately	19 (21.6)	90 (51.1)	1	1
	Within 1 h	16 (18.2)	36 (20.5)	2.105 (0.976, 4.543)	2.603 (1.87, 7.970)^a^
	After 1 h	53 (60.2)	50 (28.4)	5.021 (2.68, 9.407)	7.231 (2.753, 18.93)^a^
CPR	Yes	18 (20.5)	30 (17.0)	1.553 (0.796, 3.032)	2.858 (0.859, 9.510)
	No	70 (79.5)	146 (83.0)	1	1
Complication	Yes	27 (30.7)	42 (23.9)	1.721 (0.961, 3.032)	2.282 (0.636, 8.180)
	No	61 (69.3)	134 (76.1)	1	1
APGAR at 5 min	< 7	48 (54.5)	43 (24.4)	3.712 (2.515, 6.385)	3.712 (1.568, 8.788)^a^
	≥ 7	40 (45.5)	133 (75.6)	1	1
Type of pregnancy	Single	80 (90.9)	169 (90.0)	1	1
	Twin+	8 (9.1)	7 (4.0)	0.414 (0.145, 1182)	0.607 (0.240, 1.534)
Mode of delivery	Spontaneous	65 (73.9)	142 (80.7)	1	1
	Instrumental	9 (10.2)	19 (10.8)	0.966 (0.415, 2.251)	1.143 (0.257, 5.084)
	C/s	14 (15.9)	15 (8.5)	0.490 (0.224, 1.076)	0.638 (0.154, 2.642)
Time of born	Day	27 (30.7)	101 (57.4)	1	1
	Night	61 (69.3)	75 (42.6)	3.042 (1.768, 5.235)	6.252 (2.585, 15.123)^a^
Gestational age	< 37 weeks	27 (30.7)	20 (11.4)	3.452 (1.803, 6.611)	3.689 (1.359, 10.012)^a^
	≥ 37 weeks	61 (69.3)	156 (88.6)	1	1
Wight	< 2500 g	30 (34.1)	18 (10.2)	4.54 (2.353, 8.760)	8.513 (2.711, 26.726)
	≥ 2500 g	58 (65.9)	158 (89.8)	1	1

^a^This symbol is an indictor to show significant association with outcome variable

## Discussion

This study showed that initiation of breastfeeding was significant with neonatal hypothermia. This result is consistent with a study conducted in Zambia (2014) which showed that delayed breastfeeding initiation was 7.5 times higher risk than the immediate initiation of breastfeeding. Additionally, this study is similar to a study conducted in southern Nepal, which showed that delayed breastfeeding initiation was 1.5 times higher risk than immediate initiation breastfeeding [3, 8]. This may be due to early initiation of breastfeeding facilitated skin to skin contact immediately, prevent exposing the baby to the environment, increase follow up care to mothers for their child and delayed initiation breastfeeding prone to hypoglycemia which leads to hypothermia. The difference might be due to study setting variation and study design.

Birth weight showed a significant association with neonatal hypothermia. This finding is similar to a study conducted in Gonder, Northwest of Ethiopia (2015) presented that low birth weight was 3.7 times more likely to be hypothermic than normal weight [7]. This may be due to the fact that neonate with low birth weight has a poor subcutaneous tissue, so they had weak thermoregulation and another possible reason could be due to the surface area to body ratio is high.

Preterm babies were 3.7 times more likely to be hypothermic than term babies. This study is in line with a study conducted in Pakistan (2012) discovered that preterm babies had 3.6 times higher risk when compared with term babies [9]. The possible reason could be neonates born with preterm would have an undeveloped thermoregulation center mechanism and reduced brown adipose tissue that helps to regulate body temperature.

APGAR score at 5 min had a significant association with neonatal hypothermia. This study is in line with a study conducted in California (2011) showed that a low APGAR score at 5 min was 1.5 times higher risk to be hypothermic than a high score [10]. This could be due to the fact that asphyxia can decrease oxygen concentration then decrease metabolic rate and leads to heat loss.

Lack of skin to skin contact had a significant association with neonatal hypothermia. This study is consistent with studies conducted in Zambia (2014), Nigeria (2009) and Gonder, Northwest Ethiopia (2015) showed that those who had no skin to skin contact were 3 times more likely to be hypothermic than those who had skin to skin contact [7, 8, 11]. This might be due to neonates who were not had skin to skin contact experience increase heat loss due to conduction but mother’s external body temperature has almost similar to their womb temperature. Therefore, it’s easy to maintain their body temperature when exposing to the mother’s body temperature with skin to skin.

Time of delivery had a significant association with neonatal hypothermia. This study is in line with studies conducted in Uganda and Gonder, Northwest Ethiopia (2015) babies born during night time were 6.6 times more likely to develop hypothermia as compared to those born during daytime [7, 12]. This could be due to the fact that temperature variation between night time and daytime. Additionally, a possible reason could be due to work overload during night time as the number of staffs working in the labor room during night time is not equal to daytime staffs.

Practicing bathing within 24 h had a significant association with neonatal hypothermia. This study is in line with a study conducted in southern Nepal (2010) showed that initiated bathing after birth within 24 h was 1.2 times greater risk to be hypothermic when compared with mothers who were not practiced bathing with 24 h [3]. This discrepancy may be due to the sampling size difference. This might be due to the fact that bathing newborn within 24 h has heat lost when exposed to the cooler water, environment, and air by evaporation, conduction, and convection respectively.

### Conclusion

In this study, has found that some maternal, environmental and neonatal factors had contributed to the risk of neonatal hypothermia. Delayed of breastfeeding initiation, low birth weight, preterm, low Apgar score at 5th min, night time delivery and bathed within 24 h after born and skin to skin contact was identified as independent risk factors of neonatal hypothermia. Strengthen perinatal care of newborns and interventions babies born with complications are recommended.

## Limitation of the study

- This study is quantitative, it was better if the qualitative approach was also employed to investigate in detail on extra determinant factors of neonatal hypothermia.
- This study is conducted in one zone of the region, it was better if another zone incorporated into the study for it would have been a better generalization to the region.

## Abbreviations

APGAR: appearance, pulse, grimace, activity, and respiratory rate
AOR: adjusted odd ratio
COR: crudes odd ratio
SPSS: statistics package for social science
WHO: World Health Organization
TRHB: Tigray Regional Health Bureau

## References

[1] WHO (2010). Intervention to prevent hypothermia at birth in preterm and or low-birth weight infants.

[2] Lunze K (2013). The global burden of neonatal hypothermia: systematic review of a major challenge for newborn survival. BMC Med.

[3] Mullany LC (2010). Neonatal hypothermia and associated risk factors among newborns of southern Nepal. BMC Med.

[4] WHO (2010). Newborn care.

[5] Mullany LC (2010). Neonatal hypothermia in low-resource settings. Semin Perinatol.

[6] Liu L, Johnson HL, Cousens S, Perin J, Scott S (2012). Global, regional, and national causes of child mortality: an updated systematic analysis for 2010 with time trends since 2000. Lancet.

[7] Seyum T, Ebrahim E (2015). Proportion of neonatal hypothermia and associated factors among new-borns at Gondar University Teaching and Refferal Hospital, Northwest Ethiopia: a hospital based cross sectional study. Gen Med (Los Angel).

[8] Lunze K, Yeboah-Antwi K, Marsh DR, Kafwanda SN, Musso A (2014). Prevention and management of neonatal hypothermia in rural Zambia. PLoS ONE.

[9] Ali SR, Mirza R, Qadir M, Ahmed S, Bhatti Z, Demas S (2012). Neonatal hypothermia among hospitalized high risk newborns in a developing country. Pak J Med Sci.

[10] Miller SS, Lee HC, Gould JB (2011). Hypothermia in very low birth weight infants: distribution, risk factors and outcomes. J Perinatol.

[11] Ogunlesi TA, Ogunfowora OB, Ogundeyi MM (2009). Prevalence and risk factors for hypothermia on admission in Nigerian babies < 72 h of age. J Perinat Med.

[12] Byaruhanga R, Bergstrom A, Okong P (2005). Neonatal hypothermia in Uganda: prevalence and risk factors. J Trop Pediatr.

